# Left Ventricular Relative Wall Thickness Versus Left Ventricular Mass Index in Non-Cardioembolic Stroke Patients

**DOI:** 10.1097/MD.0000000000000872

**Published:** 2015-05-22

**Authors:** M-Sherif Hashem, Hayrapet Kalashyan, Jonathan Choy, Soon K. Chiew, Abdel-Hakim Shawki, Ahmed H. Dawood, Harald Becher

**Affiliations:** From the Jeddah Heart Institute, Erfan & Bagedo Hospital, Jeddah, Saudi Arabia (M-SH, A-HS, AHD); and Mazankowski Alberta Heart Institute, University of Alberta Hospital, Edmonton, Canada (HK, JC, SKC, HB).

## Abstract

In non-cardioembolic stroke patients, the cardiac manifestations of high blood pressure are of particular interest. Emerging data suggest that echocardiographically determined left ventricular hypertrophy is independently associated with risk of ischemic stroke.

The primary objective of this study was to evaluate the frequency of different patterns of left ventricular (LV) remodeling and hypertrophy in a group of consecutive patients admitted with non-cardioembolic stroke or transient ischemic attack (TIA). In particular, we were interested in how often the relative wall thickness (RWT) was abnormal in patients with normal LV mass index (LVMI). As both abnormal RWT and LVMI indicate altered LV remodeling, the secondary objective of this research was to study whether a significant number of patients would be missing the diagnosis of LV remodeling if the RWT is not measured.

All patients were referred within 48 hours after a stroke or a TIA for a clinically indicated transthoracic echocardiogram. The echocardiographic findings of consecutive patients with non-cardioembolic stroke or TIA were analyzed.

All necessary measurements were performed in 368 patients, who were enrolled in the study. Mean age was 63.7 ± 12.5 years, 64.4% men. Concentric remodeling carried the highest frequency, 49.2%, followed by concentric hypertrophy, 30.7%, normal pattern, 15.5%, and eccentric hypertrophy, 4.1%. The frequency of abnormal left ventricular RWT (80.4%) was significantly higher than that of abnormal LVMI (35.3%), (McNemar *P* < 0.05).

In this group of non-cardioembolic stroke patients, abnormal LV remodeling as assessed by relative wall thickness is very frequent. As RWT was often found without increased LV mass, the abnormal left ventricular geometry may be missed if RWT is not measured or reported.

## BACKGROUND

Arterial hypertension is one of the major risk factors for stroke. In up to 70% of patients admitted with a stroke, arterial hypertension is found.^1^ The fact that antihypertensive treatment has reduced morbidity and mortality from cardiovascular disease disproportionately less than blood pressure (BP) reduction,^2,3^ has led investigators to search for different cardiovascular risk factors among hypertensive patients. In further studies, echocardiographically detected left ventricular hypertrophy is defined to be independently associated with risk of ischemic stroke.^4–9^

The recommendations for chamber quantification of the American Society of Echocardiography (ASE), developed in conjunction with the European Association of Echocardiography (EAE)^10^ include a series of measurements for assessment of LV adaptation to arterial hypertension. Using measurements of LV wall thickness and LV diameter, the 3 forms of LV response to high blood pressure can be found as: concentric remodeling (abnormal relative wall thickness [RWT] and normal LV mass index [LVMI]), concentric hypertrophy (abnormal RWT and LVMI), and eccentric LV hypertrophy (abnormal LVMI and normal RWT). This classification may provide incremental value beyond ventricular mass for further cardiovascular risk stratification.^11–14^

Measurement of LV mass has been widely used to assess cardiac organ damage by arterial hypertension. However, cardiac damage can already be present in patients with normal LV mass. Concentric remodeling as detected by abnormal RWT but normal LV mass is an early form of cardiac adaptation to high blood pressure.^15^

The risk of death or the composite end point of death from cardiovascular causes, reinfarction, heart failure, and stroke is lowest for patients with normal geometry, and gradually increases with concentric remodeling, eccentric hypertrophy, and concentric hypertrophy. Increased risk associated with RWT is independent of LVMI.^14,16^

There is evidence suggesting the LV geometric patterns as risk for stroke in an ethnically mixed population.^17^ In that work, it has been shown that assessment of LV mass and RWT should be included in stroke risk evaluation.

The primary objective of this study was to evaluate the frequency of different patterns of left ventricular (LV) remodeling and hypertrophy in a group of consecutive patients admitted with non-cardioembolic stroke or transient ischemic attack (TIA). In particular, we were interested in how often the relative wall thickness (RWT) was abnormal in patients with normal LVMI. As both abnormal RWT and LVMI indicate altered LV geometry, the secondary objective of this research was to assess whether a significant number of patients would be missing the diagnosis of LV remodeling if the RWT is not measured or reported.

## METHODS

### Study Population

The data were drawn from an audit on stroke/TIA patients at the Jeddah Heart Institute where the echocardiographic examinations are routine diagnostic measures in all patients admitted to the hospital with stroke or TIA. From 2005 to 2010, the echocardiographic findings of consecutive patients were included in a database. All patients were referred from the Department of Neurology of the Erfan&Bagedo Hospital within 48 hours after a stroke or a TIA for a clinically indicated transthoracic echocardiogram. The data were analyzed in collaboration with the Mazankowski Alberta Heart Institute. The study protocol was approved by the Jeddah Heart Institute Health Research Ethics Committee.

### Exclusion Criteria

1. Patients found clinically, by history or echocardiography, to have associated findings that may lead to a cardioembolic stroke including mitral stenosis, aortic stenosis, or any possible aortic source of emboli congenital heart disease, current or history of Atrial Fibrillation, and patients known to have Chronic Obstructive Pulmonary Disease (usually limited echo window).
2. Patients who were found on computed tomography (CT) or magnetic resonance imaging (MRI) to have multiple infarcts, as this was likely to be an embolic event, even though the source could not be ascertained.
3. Patients who had ischemic cardiac events, for example, myocardial infarction (MI) or Coronary Artery Bypass Graft surgery, as an embolic source cannot be ruled out and because the formulae used for LVMI or RWT assessment would not apply due to the lack of homogeneity of wall thickness (eg, scarring).
4. Patients with suboptimal acoustic windows or technically limited parasternal windows, which made echocardiographic measurements unreliable.
5. Patients with hemorrhagic stroke.

### Diagnostic Assessments

The diagnosis of TIA was made clinically as acute focal neurological deficits highly suspected by the experienced neurologist to be of vascular origin or caused by brain ischemia and resolved within 24 hours. Those patients should have either a normal brain imaging or no lesion, which could be a potential cause for the neurological symptoms. The stroke was defined clinically and confirmed by imaging. The initial brain CT was performed in all patients. In the majority of acute stroke patients, with no initial CT changes, the diagnosis was confirmed either by diffusion-weighted MRI or by repeat CT after 24 hours from time of symptoms onset. By chart review and history, all patients were reviewed for the presence of the traditional stroke risk factors: hypertension, diabetes mellitus, and obesity. The diagnosis of arterial hypertension was based on patients’ medical history, presence of treatment or blood pressure ≥140/90 mmHg.^18,19^ Diabetes mellitus (DM) was diagnosed as abnormal fasting glucose results, history of the disease, or presence of appropriate treatment.^20^ Obesity was defined according to WHO criteria as a body mass index^21^ >30 kg/m^2^. Serum cholesterol levels and smoking were not included into the database, as they were reported to have no impact on LV hypertrophy.^22^

### Echocardiography

Transthoracic echocardiography was performed within 48 hours after stroke or TIA using HDI 5000 ultrasound (Bothell, WA), equipped with 3 MHz transducer. Parasternal and apical 2-dimensional echocardiograms (2D) were acquired according to the ASE/EAE recommendations.^10^ In end diastole, the septum wall thickness (SWTd), posterior LV wall thickness (PWTd), and the diameter of the left ventricle (LVIDd) were measured on the 2D echo recordings.

LVMI was calculated using the following equations:

LV mass = 0.8 (1.04 [LVID + PWTd + SWTd]^3^ – [LVID]^3^) + 0.6 g

LVMI = LVM/body surface area

RWT was calculated by dividing the sum of SWTd and PWTd by the LVIDd. According to the ASE/EAE, RWT of 0.22 to 0.42 was regarded as normal.

The reference ranges used to define abnormal left ventricular thickness were:

RWT (male and female) ≤0.42

LVMI (male) <115 g/m^2^

LVMI (female) <95 g/m^2^

The geometric changes of the left ventricle were classified based on left ventricular mass index and relative wall thickness.^10–13^ Four different groups were defined: elevated RWT with increased LVMI identified as concentric hypertrophy, elevated RWT with normal LVMI as concentric remodeling, normal RWT with increased LVMI as eccentric remodeling, and normal geometry.

All echocardiograms were performed and analyzed by an experienced cardiologist. An off-site read of randomly selected echocardiographic readings was performed at the Mazankowski Alberta Heart Institute. The reproducibility of RWT measurements was assessed in 15 randomly selected patients using interclass correlation coefficient, *R* statistic = 0.8 (95% confidence interval, 2-tailed *P* < 0.05).

### Statistics

Data were stored and analyzed by IBM-SPSS (Version 22; IBM Corp, Armonk, NY) statistical software. The frequency of different types of LV wall abnormality was assessed using the descriptive statistics. To assess the association of the risk factors with LV remodeling, the Generalized Linear Models have been employed. The significance of the difference between abnormal RWT and LVMI was assessed using McNemar test.

## RESULTS

A total of 870 patients with stroke and TIA underwent transthoracic echocardiography in the period from January 2005 to July 2010. Four hundred ninety-one (491) of them were likely to have cardioembolic mechanism of stroke or TIA or other exclusion criteria, including old MI. Three hundred seventy-nine (379) subjects matched the inclusion and exclusion criteria. Eleven patients had suboptimal echo windows. All necessary measurements were performed in 368 patients, who were enrolled in the study. The study subjects were of a multiethnic cohort in whom Arab ethnicity was 52.4%. The rest were of North African, African, Central Asian, Indian, or Far East origins. Mean age was 63.7 ± 12.5 (SD). The sex distribution was as follows: 237 males (64.4%) and 131 females.02 (35.6%) patients.

The frequencies of risk factors and abnormal RWT and LVMI based on sex distribution are presented in Table 1.

**TABLE 1:**
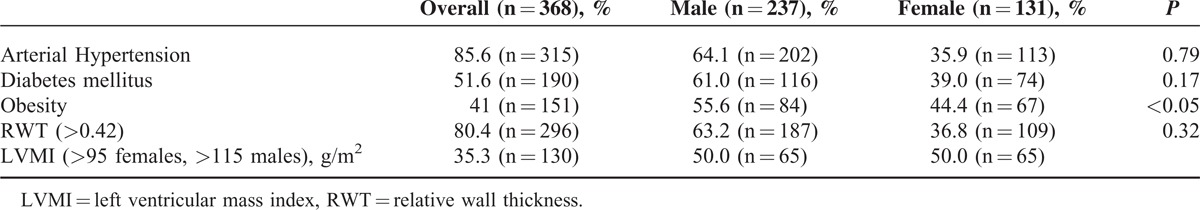
The Frequencies of the Risk Factors and Increased RWT and LVMI in the Study Group **(***P* Value Refers to Statistical Differences Between Male and Female)

The frequencies of different patterns of LV remodeling are distributed as follows: concentric remodeling carried the highest frequency (49.2%), followed by concentric hypertrophy (30.7%), normal pattern (15.5%), and eccentric hypertrophy (4.1%). The effects of main risk factors on different types of LV remodeling are presented in Table 2.

**TABLE 2:**
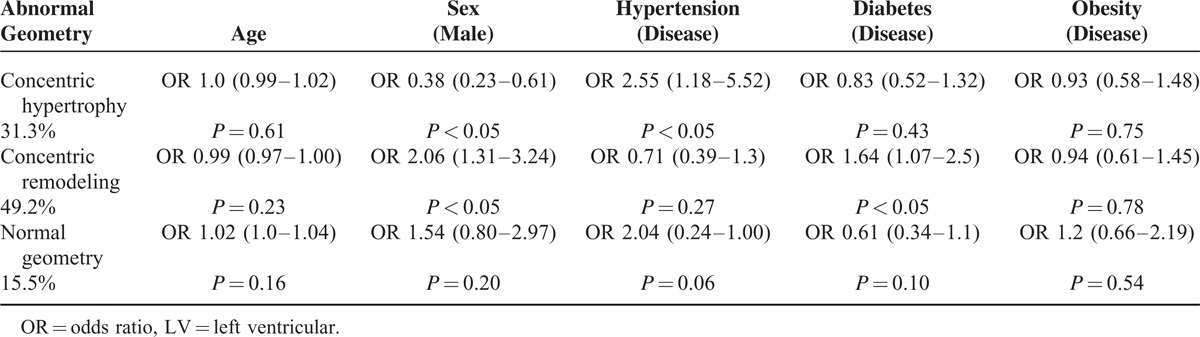
The Association of Risk Factors With the Different Types of LV Remodeling

The frequency of abnormal RWT was significantly higher than that of abnormal LVMI, McNemar *P* < 0.05 (Table 3).

**TABLE 3:**
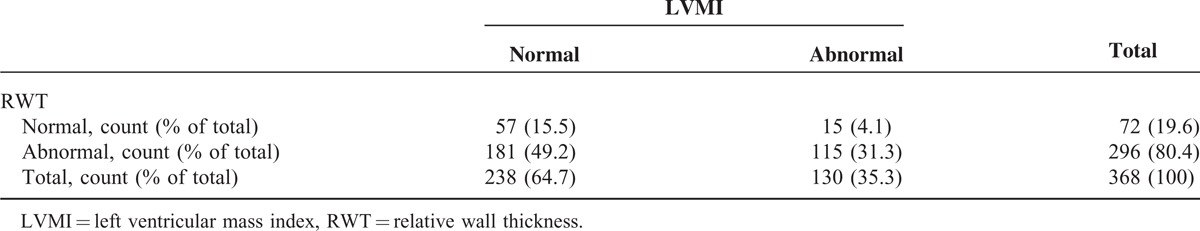
RWT and LVMI Cross-tabulation

## DISCUSSION

The initiative for this study came from the very frequent observation of abnormal RWT as a salient finding in patients referred for echocardiography at the Jeddah Heart Institute after stroke. This is the largest series of consecutive stroke and TIA patients, in whom the frequency of RWT and other parameters of LV remodeling is reported. The results of our study suggest a very high frequency of LV remodeling subtypes in this multiethnic patient's cohort with non-cardioembolic stroke. Our results suggest that isolated RWT (concentric remodeling) was independently associated with diabetes in men, and concentric hypertrophy was associated with hypertension in women. The eccentric hypertrophy is not included in the table due to very small sample size (15 patients).

The message of our study is that LV remodeling as assessed by abnormal RWT has a significantly higher frequency than increased LVMI in non-cardioembolic stroke patients and indicates abnormal LV geometry expressed by concentric remodeling. When physicians look for cardiac remodeling, they tend to concentrate on LV mass and often neglect RWT. However, abnormal RWT was often found with normal LV mass. Abnormal RWT combined with normal LV mass (concentric remodeling) is clinically relevant, for example, in patients who present with borderline hypertension, elevated blood pressure only at the admission, and/or no known history of arterial hypertension. In these patients, antihypertensive treatment needs to be considered.^22,23^

In Di Tullio's study, abnormal RWT significantly increased the stroke risk and no interaction was detected between RWT and LVMI with regard to stroke risk. In their study, Eguchi et al^16^ concluded that in hypertensive subjects with DM type 2, echocardiographic RWT is a predictor of cardiovascular events independent of LVMI and other confounders.

LVMI has been widely used to assess LV hypertrophy as a consequence of arterial hypertension and reported as an independent risk factor for ischemic stroke.^24–27^ LV mass has been regarded as the best measure of LV hypertrophy and is used in many studies using echocardiography or MRI. Echocardiography is more frequently used in larger studies on hypertension because of its lower costs, but the reproducibility of echocardiographic measurements of LV mass is limited.^28–30^ Although LVMI calculation is performed using the same linear measurements as RWT, small errors in measurements have a high impact on the calculation as the equation uses squared values. In addition, as the LV diameter depends on the body mass, RWT appears to be a useful simple measurement to assess LV hypertrophy in patients with different body conditions and should not be neglected. It represents a ratio, unlike LVMI which is an absolute value; RWT may thus be less vulnerable to variability related to weight and height.

The isolated finding of abnormal RWT indicates LV remodeling, which is an early feature of hypertensive heart disease. Because of the significantly higher frequency of abnormal RWT in this study, it is important that physicians who look after patients with stroke pay attention to this value on the echocardiography reports. The detection of cardiac abnormalities such as remodeling during the acute stroke phase may result in additional diagnostics for assessment of hypertension. This is particularly important when the blood pressure is normal during admission or when the patient has no history of hypertension. In patients with elevated blood pressure, the finding of LV remodeling makes the diagnosis of chronic hypertension more likely than accidental (hypertension related to the admission). Therefore, considering RWT for medical treatment is likely to result in better care of patients. Considering the high frequency of abnormal RWT with normal LV mass in the reported group of stroke patients, outcome studies are warranted to answer the question whether patients with abnormal RWT would benefit from medical treatment as patients with increased LV mass do.

### Study Limitations

The study was conducted with emphasis on echocardiographic features of non-cardioembolic stroke patients and mainly designed to analyze the prevalence of RWT in this group. Echocardiographic criteria used to define LV hypertrophy in available studies are not uniform^31^ and vary substantially. Therefore, comparison with other studies is limited.

It is well known that the diagnosis of TIA is purely clinical. There is a poor agreement even between the stroke specialists in differentiating TIAs from mimics.^32^ So, this shortcoming could not be avoided in our study and the reader should consider that a proportion of our study group may represent mimics.

## CONCLUSION

In this group of consecutive patients with non-cardioembolic stroke, abnormal LV geometry as assessed by RWT is very frequent. As abnormal RWT was often found with normal LVMI, abnormal left ventricular geometry may be missed if RWT is not measured or reported.
